# Distinction and quantification of carry-over and sample interaction in gas segmented continuous flow analysis

**DOI:** 10.1155/S1463924697000254

**Published:** 1997

**Authors:** Jia-Zhong Zhang

**Affiliations:** Cooperative Institute for Marine and Atmospheric Studies Rosenstiel School of Marine and Atmospheric Science/AOML NOAA University of Miami Miami Florida FL33149 USA

## Abstract

The formulae for calculation of carry-over and sample interaction are derived for the first time in this study. A scheme proposed by Thiers et al. (two samples of low concentration followed by a high concentration sample and low concentration sample) is verified and recommended for the determination of the carry-over coeffcient. The derivation demonstrates that both widely used schemes of a high concentration sample followed by two low concentration samples, and a low concentration sample followed by two high concentration samples actually measure the sum of the carry-over coeffcient and sample interaction coefficient. A scheme of three low concentration samples followed by a high concentration sample is proposed and verified for determination of the sample interaction coeffcient. Experimental results indicate that carry-over is a strong function of cycle time and a weak function of ratio of sample time to wash time. Sample dispersion is found to be a function of sample time. Fitted equations can be used to predict the carry-over, absorbance and dispersion given sample times, and wash times for an analytical system. Results clearly show the important role of intersample air segmentation in reducing carry-over, sample interaction and dispersion.


Journal of Automatic Chemistry, Vol. 19, No. 6 (November-December 1997) pp. 205-212

### Distinction and quantification of carry-over and sample interaction in gas segmented continuous flow analysis

**Jia-Zhong Zhang**

*Cooperative Institute for Marine and Atmospheric Studies, Rosenstiel School of Marine and Atmospheric Science/AOML, NOAA, University of Miami, Miami, Florida FL33149, USA*

*The formulae for calculation of carry-over and sample interaction are derived for the first time in this study. A scheme proposed by Thiers* et al. *(two samples of low concentration followed by a high concentration sample and low concentration sample) is verified and recommended for the determination of the carry-over coefficient. The derivation demonstrates that both widely used schemes of a high concentration sample followed by two low concentration samples, and a low concentration sample followed by two high concentration samples actually measure the sum of the carry-over coefficient and sample interaction coefficient. A scheme of three low concentration samples followed by a high concentration sample is proposed and verified for determination of the sample interaction coefficient. Experimental results indicate that carry-over is a strong function of cycle time and a weak function of ratio of sample time to wash time. Sample dispersion is found to be a function of sample time. Fitted equations can be used to predict the carry-over, absorbance and dispersion given sample times, and wash times for an analytical system. Results clearly show the important role of intersample air segmentation in reducing carry-over, sample interaction and dispersion.*

### Introduction

In gas segmented continuous flow analysis, there are two different types of segmentation gas bubbles. The first type is usually injected by a pump tube or bubble injector into the first reagent, in order to segment the reagent stream at the beginning of flow. A surfactant is generally added to the first reagent solution to reduce the surface tension between the fluid stream and inner wall of tubing. This gas segmented reagent stream merges with the other reagents and samples as it flows through the analytical manifold. The samples are introduced to the flow stream by an autosampler. The sampler probe is designed to reside consecutively between the sample cups and wash reservoir, resulting in a series of sample slugs aspirated from different sample cups separated by wash water slugs. Because pumping is continuous while the sampler probe moves between a sample cup and wash reservoir, an air bubble is aspired into the sample stream at the interface between sample slugs and wash water slugs. If the sampler is set at 'pecking' mode, more than one intersample air bubble will be inserted at the interface which enhances the separation between the sample and wash solution. This is known as 'intersample air segmentation' (ISAS).

The intersample air bubble is initially at atmospheric pressure, but when it passes under the pump plate it is compressed by the pump roller. Constant flow rates in peristaltic pumps depend upon the incompressibility of the solution. In this case the sample flow rate will be momentarily decreased while the intersample bubble passes the pump roller. Intersample air compression results in a notching at the top of the absorbance peak. Since the size of an intersample air bubble is proportional to the inner diameter of the sample pump tube, interference from intersample air compression becomes appreciable when a large sample pump tube is used to increase the analytical sensitivity. To solve this problem, debubbling the sample line to remove the intersample air segmentation has become a common procedure.

Intersample bubbles are aspired through the sample probe from ambient air and they are incompatible with some analyses. In trace ammonia analysis, for example, intersample air bubbles must be completely removed to avoid ammonia contamination from ambient air and high-purity grade nitrogen is used as a segmentation gas.

Although it has been known for many years that segmentation gas bubbles [1,2] reduce carry-over, sample interaction and dispersion, the relative importance of intersample air segmentation to overall reagent gas segmentation has not been fully appreciated. The aim of this study was to quantify the contribution of intersample air segmentation in reducing carry-over, sample interaction and sample dispersion.

To quantify the effect of intersample air segmentation it is necessary to individually quantify carry-over, sample interaction and sample dispersion within the analytical system. Although gas segmented continuous flow analysis has played an important role in automated chemical analysis since the 1950s [3], there have been inconsistencies in the definitions of carry-over and sample interaction, hence the procedures used for quantification and rectification.

Thiers *et al.* were the first to study the carry-over, which they called 'interaction between samples', in continuous flow analyses in 1964 [4]. They proposed an 'interaction test pattern', which used a series of cups containing standards arranged in the concentration sequence 'zero, low, high, repeat of low' [4, 5]. The absolute 'interaction' in concentration units was calculated from the difference between the apparent concentration of the second low standard and first low standard. This value, divided by that of the high standard, defined the degree of interaction. They demonstrated that so-called interaction was directly proportional to the concentration of the preceding sample and was independent of the concentration of

0142-0453/97 $12·00 © 1997 Taylor & Francis Ltd the measured sample. They also pointed out that any given sample interacts only with the sample immediately following it and not vice versa. In 1967 Thiers *et al.* proposed the use of two kinetic parameters, the half wash time and lag phase time, to characterize the interaction between samples in flow systems [6,7].

In 1969 the Laboratory Equipment and Methods Advisory Group (Broughton *et al.*) [8] first used the term 'carry-over', to denote the influence of the concentration of an analyte in one sample upon the result obtained for the following sample. They recommended a scheme for correction of carry-over by analysing three identical samples of a high concentration followed by three identical samples of a low concentration. A carry-over coefficient was defined as the ratio of difference between the first low concentration sample and the third low concentration sample to the difference between the third high concentration sample and third low concentration sample. They also defined a true peak height as that achieved when the sample is measured consecutively several times. Since such a peak contains carry-over from a preceding peak of similar peak height, this definition is problematical. In 1970, Walker *et al.* [9] confirmed and extended Thiers *et al.*'s work [6]. Preferring the term 'carry-over' instead of 'interaction between samples', Walker *et al.* defined the true peak height as the one preceded by a blank. Today, carry-over is a well accepted term referring to the effect of concentration of a preceding sample upon a subsequent sample. Carry-over is an important parameter in monitoring system performance. The formulae or schemes, however, used to quantify carry-over are still inconsistent. For example, the Softpac™ software supplied with the Alpkem autoanalyser [10] uses a high concentration sample followed by two low concentration samples to measure the carry-over. The Fastpac™ software (Window version 1.3) [11] offered by the same company changed this to a low concentration sample followed by two high concentration samples. Version 1.31 of the same Fastpac™ software [12] switched back to a high concentration sample followed by two low concentration samples. In the software TAOS supplied by Bran + Luebbe [13], carry-over is measured using a high concentration sample followed by two low concentration samples. Inconsistencies may be partly due to the fact that the formulae or schemes used for carry-over correction have never been proved by mathematical derivation. No effort seems to have been made to distinguish between carry-over and sample interaction. It is essential to clarify the definition of carry-over and sample interaction, and to derive correct formulae for their calculations, in order to rigorously investigate the role of intersample air segmentation.

### Definitions and derivations

### *Carry-over*

Carry-over is a phenomenon in which the analyte in a given sample is 'carried' by an analytical system 'over' to the following sample. Carry-over is inherently unidirectional, i.e. each sample can affect the sample behind it but never the one in front. The magnitude of carry-over depends on the concentration of the preceding sample, not the concentration of the measured sample nor the difference between the preceding and measured sample. Carry-over should be subtracted from measured sample peak height to obtain a true sample peak height.

Angelova and Holy [14] have shown that, for a system with a linear calibration graph, the carry-over signal for a given sample is linearly dependent upon the absorbance of the preceding sample. For a given sample $i$, therefore, the amount of carry-over, CO, is proportional to the absorbance of the preceding sample, $A_{i-1}$:

$$\mathrm{CO} = k_{\mathrm{co}} A_{i-1} \quad (1)$$

where $k_{\mathrm{co}}$ is the carry-over coefficient. To correct the carry-over effect, CO must be subtracted from measured absorbance of a given sample, $A_i$:

$$A_{i,c} = A_i - k_{\mathrm{co}} A_{i-1} \quad (2)$$

where $A_{i,c}$ is the corrected absorbance of sample $i$.

### *Sample interaction*

Sample interaction refers to mixing between adjacent samples. Instead of a unidirectional effect, it is a reciprocal one. For example, both samples $i-1$ (front) and $i+1$ (behind) could interact with sample $i$. Sample interaction becomes appreciable when two samples pass through an unsegmented tube or a sample line is separated by little or no wash. Thiers *et al.* [6] used the term 'interaction between samples' to refer to carry-over. True sample interaction has not been studied in continuous flow analysis. Sample interaction, SI, can be assumed to be proportional to the differences in the concentrations of analyte between adjacent samples:

$$\mathrm{SI} = k_{\mathrm{si}}(A_i - A_{i-1}) + k_{\mathrm{si}}(A_i - A_{i+1}) \quad (3)$$

where $k_{\mathrm{si}}$ is the sample interaction coefficient. To correct the effect of sample interaction, SI must be added to the absorbance of a given sample $i$, $A_i$

$$A_{i,c} = A_i + k_{\mathrm{si}}(A_i - A_{i-1}) + k_{\mathrm{si}}(A_i - A_{i+1}) \quad (4)$$

The sample interaction correction is negative if the absorbances of adjacent samples, $A_{i-1}$ or $A_{i+1}$, are greater than $A_i$.

To obtain true peak height, it is necessary to correct for both carry-over and sample interaction:

$$A_{\mathrm{i,c}} = A_i - k_{\mathrm{co}} A_{i-1} + k_{\mathrm{si}}(A_i - A_{i-1}) + k_{\mathrm{si}}(A_i - A_{i+1}) \quad (5)$$

where $A_{i-1}$, $A_i$ and $A_{i+1}$ are measured absorbances of sample $i-1$, $i$ and $i+1$, respectively. In other words, to obtain absolute absorbances, the coefficients for carry-over and sample interaction, $k_{\mathrm{co}}$ and $k_{\mathrm{si}}$, must be determined for the specific analytical system.

*The determination of* $k_{co} + k_{si}$: The most widely used scheme to measure the carry-over coefficient, $k_{\mathrm{co}}$ is arranged in a sequence of a high analyte concentration sample followed by three identical low (zero or near zero) concentration samples (scheme HLLL, $A_{i-1} \gg A_i \sim A_{i+1} \sim A_{i+2}$ as shown in figure 1). For sample $i$, which is the first low concentration sample, equation (5) can be simplified to:

$$A_{i,c} = A_i - k_{\mathrm{co}} A_{i-1} + k_{\mathrm{si}}(A_i - A_{i-1}) \quad (6)$$

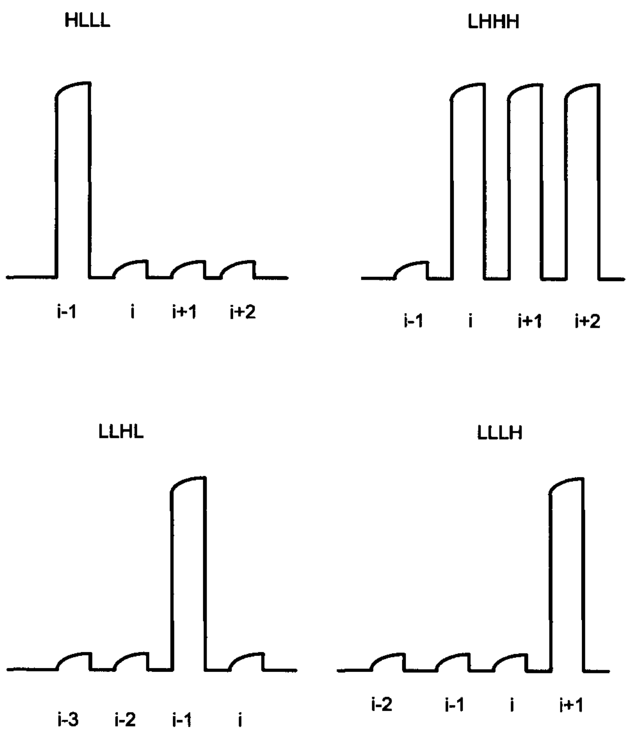

*Figure 1. Output signals of four different schemes used to measure carry-over and sample interaction coefficients.*

Since $A_i \ll A_{i-1}$, equation (6) can be further simplified to:

$$A_{i,c} = A_i - k_{co}A_{i-1} - k_{si}A_{i-1} \tag{7}$$

For sample $i + 1$, which is the second zero concentration sample, the correction should be made as follows:

$$A_{i+1,c} = A_{i+1} - k_{co}A_i + k_{si}(A_{i+1} - A_i) + k_{si}(A_{i+1} - A_{i+2}) \tag{8}$$

Since $A_{i+1}$, $A_i$ and $A_{i+2}$ are all close to zero, equation (8) can be simplified to:

$$A_{i+1,c} = A_{i+1} \tag{9}$$

Since $i$ and $i + 1$ have the same concentration, corrected absorbances should be the same, i.e.:

$$A_{i,c} = A_{i+1,c} \tag{10}$$

Substituting equations (9) and (10) into (7) yields:

$$A_i - k_{co}A_{i-1} - k_{si}A_{i-1} = A_{i+1} \tag{11}$$

Rearranging equation (11) gives:

$$k_{co} + k_{si} = (A_i - A_{i+1})/A_{i-1} \tag{12}$$

If a low concentration standard instead of zero or near zero standards is used for L in an HLLL scheme, then the assumption of $A_{i-1} \gg A_i$ is not valid. By taking the difference between the high standard ($A_{i-1}$) and the first low standard ($A_i$) into account, a similar equation to (12) can be derived:

$$k_{co} + k_{si} = (A_i - A_{i+1})/(A_{i-1} - A_i) \tag{13}$$

It is evident from equations (12) or (13) that this popular scheme really measures the sum of $k_{co} + k_{si}$.

Another equally popular scheme used to measure the carry-over coefficient, $k_{co}$, is a low (zero or near zero) analyte concentration sample followed by three identical high concentration samples (scheme LHHH, $A_{i-1} \ll A_i \sim A_{i+1} \sim A_{i+2}$, as shown in figure 1). For sample $i$, which is the first high concentration sample, equation (5) can be simplified to:

$$A_{i,c} = A_i + k_{si}(A_i - A_{i-1}) \tag{14}$$

Since $A_i \gg A_{i-1}$ equation (14) can be further simplified to:

$$A_{i,c} = A_i + k_{si}A_i \tag{15}$$

For sample $i + 1$, which is the second high concentration sample, the correction should be made as follows:

$$A_{i+1,c} = A_{i+1} - k_{co}A_i + k_{si}(A_{i+1} - A_i) + k_{si}(A_{i+1} - A_{i+2}) \tag{16}$$

Since $A_{i+1}$, $A_i$ and $A_{i+2}$ are of similar absorbances, the differences between them are close to zero. Equation (16) can be simplified to:

$$A_{i+1,c} = A_{i+1} - k_{co}A_i \tag{17}$$

Since $i$ and $i + 1$ have the same concentrations, corrected absorbances should be the same, i.e.:

$$A_{i,c} = A_{i+1,c} \tag{18}$$

Substituting equations (17) and (18) into (15) yields:

$$A_i + k_{si}A_i = A_{i+1} - k_{co}A_i \tag{19}$$

Rearranging equation (19) gives:

$$k_{co} + k_{si} = (A_{i+1} - A_i)/A_i \tag{20}$$

The derivations indicate that this scheme also measures the sum of $k_{co} + k_{si}$. Neither can partition the theoretically independent values of $k_{co}$ and $k_{si}$. To separate $k_{co}$ or $k_{si}$ from the sum, the value of $k_{co}$ or $k_{si}$ must be obtained independently.

*The determination of* $k_{co}$: Although the scheme of 'interaction test pattern' originally proposed by Thiers *et al.* [5] is not in fashion, nor is it used in the commercial software supplied with autoanalysers, the following derivation will verify that it, in fact, is the sole correct scheme for determining carry-over coefficient, $k_{co}$.

Thiers *et al.*'s [5] scheme uses a series of cups containing standards arranged in the concentration sequence of low, low, high, repeat of low as shown in the scheme of LLHL in figure 1. If the third low standard is denoted as sample $i$, then $A_{i-2} \ll A_{i-1} \gg A_i$. For sample $i$, equation (5) can be simplified to:

$$A_{i,c} = A_i - k_{co}A_{i-1} + k_{si}(A_i - A_{i-1}) \tag{21}$$

Since $A_i \ll A_{i-1}$ equation (21) can be further simplified to:

$$A_{i,c} = A_i - k_{co}A_{i-1} - k_{si}A_{i-1} \tag{22}$$

For sample $i - 2$, which is the second low concentration sample, the correction should be made as follows:

$$A_{i-2,c} = A_{i-2} - k_{co}A_{i-3} + k_{si}(A_{i-2} - A_{i-3}) + k_{si}(A_{i-2} - A_{i-1}) \tag{23}$$

Since $A_{i-3}$ and $A_{i-2}$ are close to zero, the difference between them is negligible. Equation (23) can be simplified to:

$$A_{i-2,c} = A_{i-2} - k_{si}A_{i-1} \quad (24)$$

Since $i$ and $i-2$ have the same concentration, corrected absorbances should be the same, i.e.:

$$A_{i,c} = A_{i-2,c} \quad (25)$$

Combining equations (22), (24) and (25) yields:

$$A_i - k_{co}A_{i-1} - k_{si}A_{i-1} = A_{i-2} - k_{si}A_{i-1} \quad (26)$$

Rearranging equation (26) gives:

$$k_{co} = (A_i - A_{i-2})/A_{i-1} \quad (27)$$

It is evident from equation (27) this scheme can measure the carry-over coefficient, $k_{co}$. $k_{si}$ should be obtained by subtracting this value from the sum of $k_{co} + k_{si}$ derived from the schemes HLLL or LHHH.

*The determination of* $k_{si}$: In this study a scheme is proposed to measure the sample interaction coefficient, $k_{si}$ using a similar approach. This scheme uses three identical low (zero or near zero) concentration samples followed by a high concentration sample (scheme LLLH, $A_{i-2} \sim A_{i-1} \sim A_i \ll A_{i+1}$, shown in figure 1). For sample $i$, which is the third low concentration sample, equation (5) can be simplified to:

$$A_{i,c} = A_i + k_{si}(A_i - A_{i+1}) \quad (28)$$

For sample $i-1$, which is the second zero concentration sample, the correction should be made as follows:

$$A_{i-1,c} = A_{i-1} - k_{co}A_{i-1} + k_{si}(A_{i-1} - A_{i-2}) + k_{si}(A_{i-1} - A_i) \quad (29)$$

Since $A_{i-1}$, $A_i$ and $A_{i-2}$ are close to zero, equation (29) can be simplified to:

$$A_{i-1,c} = A_{i-1} \quad (30)$$

Since $i$ and $i-1$ have the same concentration, corrected absorbances should be the same, i.e.:

$$A_{i,c} = A_{i-1,c} \quad (31)$$

Substituting equations (30) and (31) into (28) yields:

$$A_{i-1} = A_i + k_{si}(A_i - A_{i+1}) \quad (32)$$

Rearranging equation (32) gives:

$$k_{si} = (A_i - A_{i-1})/(A_{i+1} - A_i) \quad (33)$$

It is evident from equation (33) that this scheme measures only $k_{si}$. The value of $k_{co}$ can be obtained by subtracting $k_{si}$ from the sum of $k_{co} + k_{si}$ if the scheme of HLLL or LHHH mentioned above is used.

### Experimental

To verify this theoretical approach, an Alpkem Flow Solution autoanalyser was used to measure the coefficients for carry-over and sample interaction in dissolved silicate analysis. The analytical procedures and methodologies used in the analysis of silicate are essentially similar to those described by Armstrong *et al.* [15], with the modifications described in Hansen *et al.* [16]. A $\beta$-molybdosilicic acid is formed by reaction of the silicate contained in the sample with molybdate in an acidic solution. The $\beta$-molybdosilicic acid is then reduced by ascorbic acid to form molybdenum blue. The absorbance of molybdenum blue, measured at 660 nm, is linearly proportional to the concentration of silicate in the sample, with a detection limit of 0·05 μM. All reagents were of analytical grade. Sodium hexafluorosilicate was used to prepare silicate standards. The Alpkem Softpac computer software was used to collect the raw data.

The coefficients for carry-over and sample interaction were measured using different schemes mentioned above. The sums of $k_{co} + k_{si}$ were determined using both the HLLL and LHHH schemes. The $k_{co}$ value was determined by the LLHL scheme. The $k_{si}$ value was determined by the LLLH scheme. Each measurement was made in seven replicates to obtain a statistically significant average.

The effects of sample time, wash time and ratio of sample time to wash time upon carry-over, sample interaction and sample dispersion were measured. Sample times and wash times were in the range of 15–80 s, therefore the cycle times (sample time plus wash time) varied from 30 to 100 s and the ratio of sample time to wash time varied from 0·25 to 4.

The first set of experiments was designed to investigate carry-over, sample interaction and sample dispersion in a system with intersample air segmentation. By adding a debubbler in the sample line at the exit of sampler, the second set of experiments was designed to investigate carry-over, sample interaction and sample dispersion in a system without intersample air segmentation. Comparing the results obtained from these two systems rigorously defines the role of intersample air segmentation.

### Results and discussion

The results of measurements (average ± standard deviation) with intersample air segmentation are summarized in table 1, and those without intersample air segmentation are summarized in table 2. Sample interaction is negligible ($k_{si} = 0$) in a system with intersample air segmentation, as shown in table 1. Without intersample air segmentation, however, sample interaction becomes appreciable ($k_{si}$ *ca.* 0·05–0·5) at cycle times shorter than 40 s, as shown in table 2. Only at cycle times greater than 40 s is sample interaction negligible ($k_{si} = 0$) in a system without intersample air segmentation. Only under the conditions of negligible sample interaction do the schemes of HLLL and LHHH measure the carry-over coefficient.

In general, carry-over coefficients, $k_{co}$, decreased with increasing cycle time. However, at a given cycle time carry-over coefficients, $k_{co}$, decreased as the sample time increased in the system with intersample air segmentation, but increased as the sample time increased in the system without intersample air segmentation. To minimize carry-over at a given cycle time, therefore, longer sample times should be used in a system with intersample

*Table 1. Carry-over, sample interaction and dispersion in a system with intersample air segmentation.*

| *Cycle time(s)* | *Sample time(s)* | *Wash time(s)* | *Ratio (s/w)* | $k_{co} + k_{si}$ *(HLLL)* | $k_{co} + k_{si}$ *(LHHH)* | $k_{co}$ *(LLHL)* | $k_{si}$ *(LLLH)* | *RSD (%)* | *Norm. Abs.* |
|---|---|---|---|---|---|---|---|---|---|
| 30 | 15 | 15 | 1 | 5·99 ± 0·44 | 3·48 ± 1·32 | 5·89 ± 0·43 | 0 | 1·42 | 0·0498 |
| 40 | 15 | 25 | 0·6 | 2·90 ± 0·25 | 0·42 ± 0·80 | 2·93 ± 0·34 | 0 | 1·31 | 0·0497 |
| 40 | 20 | 20 | 1 | 2·32 ± 0·24 | 1·95 ± 0·59 | 2·25 ± 0·29 | 0 | 1·15 | 0·0523 |
| 40 | 25 | 15 | 1·67 | 1·68 ± 0·15 | 1·99 ± 0·81 | 1·63 ± 0·16 | 0 | 1·04 | 0·0535 |
| 60 | 20 | 40 | 0·5 | 0·74 ± 0·07 | 0·20 ± 1·10 | 0·74 ± 0·04 | 0 | 0·72 | 0·0513 |
| 60 | 30 | 30 | 1 | 0·49 ± 0·04 | 0·70 ± 0·73 | 0·49 ± 0·02 | 0 | 0·65 | 0·0559 |
| 60 | 40 | 20 | 2 | 0·35 ± 0·08 | 0·33 ± 0·16 | 0·38 ± 0·08 | 0 | 0·57 | 0·0577 |
| 80 | 20 | 60 | 0·33 | 0·18 ± 0·06 | 0·66 ± 0·89 | 0·19 ± 0·04 | 0 | 0·69 | 0·0509 |
| 80 | 30 | 50 | 0·6 | 0·17 ± 0·03 | 0·69 ± 1·21 | 0·18 ± 0·04 | 0 | 0·63 | 0·0536 |
| 80 | 40 | 40 | 1 | 0·15 ± 0·04 | 0·15 ± 0·11 | 0·16 ± 0·03 | 0 | 0·16 | 0·0556 |
| 80 | 50 | 30 | 1·67 | 0·16 ± 0·04 | 0·12 ± 0·40 | 0·15 ± 0·03 | 0 | 0·53 | 0·0574 |
| 80 | 60 | 20 | 3 | 0·13 ± 0·05 | 0·37 ± 1·00 | 0·13 ± 0·04 | 0 | 0·48 | 0·0586 |
| 100 | 20 | 80 | 0·25 | 0·09 ± 0·03 | −0·24 ± 0·95 | 0·10 ± 0·02 | 0 | 0·79 | 0·0507 |
| 100 | 50 | 50 | 1 | 0·04 ± 0·02 | −0·13 ± 0·84 | 0·07 ± 0·03 | 0 | 0·58 | 0·0571 |
| 100 | 80 | 20 | 4 | 0·03 ± 0·03 | 0·10 ± 0·59 | 0·05 ± 0·04 | 0 | 0·45 | 0·0592 |

*Table 2. Carry-over, sample interaction and dispersion in a system without intersample air segmentation.*

| *Cycle time(s)* | *Sample time(s)* | *Wash time(s)* | *Ratio (s/w)* | $k_{co} + k_{si}$ *(HLLL)* | $k_{co} + k_{si}$ *(LHHH)* | $k_{co}$ *(LLHL)* | $k_{si}$ *(LLLH)* | *RSD (%)* | *Norm. Abs.* |
|---|---|---|---|---|---|---|---|---|---|
| 40 | 15 | 25 | 0·6 | 24·6 ± 3·0 | 21·21 ± 4·7 | 22·7 ± 0·5 | 0·05 ± 0·14 | 2·37 | 0·0188 |
| 40 | 20 | 20 | 1 | 28·3 ± 7·2 | 23·0 ± 1·4 | 23·0 ± 2·0 | 0·26 ± 0·34 | 2·17 | 0·0251 |
| 40 | 25 | 15 | 1·67 | 29·3 ± 5·8 | 19·1 ± 3·1 | 24·3 ± 1·2 | 0·5 ± 0·6 | 2·38 | 0·0314 |
| 60 | 20 | 40 | 0·5 | 4·87 ± 0·58 | 2·21 ± 3·26 | 4·90 ± 0·56 | 0 | 2·06 | 0·0237 |
| 60 | 30 | 30 | 1 | 5·91 ± 0·47 | 3·04 ± 2·33 | 6·16 ± 0·46 | 0 | 1·37 | 0·0335 |
| 60 | 40 | 20 | 2 | 7·79 ± 0·69 | 4·41 ± 0·61 | 7·34 ± 0·54 | 0 | 0·88 | 0·0391 |
| 80 | 20 | 60 | 0·33 | 0·72 ± 0·03 | 0·20 ± 1·68 | 0·85 ± 0·08 | 0 | 1·92 | 0·0234 |
| 80 | 40 | 40 | 1 | 1·04 ± 0·25 | 1·03 ± 1·48 | 1·02 ± 0·37 | 0 | 1·32 | 0·0417 |
| 80 | 60 | 20 | 3 | 2·05 ± 0·18 | 1·51 ± 0·34 | 2·08 ± 0·06 | 0 | 0·69 | 0·052 |
| 100 | 20 | 80 | 0·25 | 0·10 ± 0·05 | −0·64 ± 1·77 | 0·08 ± 0·4 | 0 | 0·85 | 0·0223 |
| 100 | 50 | 50 | 1 | 0·14 ± 0·08 | −0·05 ± 1·32 | 0·12 ± 0·01 | 0 | 0·83 | 0·0492 |
| 100 | 80 | 20 | 4 | 0·43 ± 0·11 | 0·11 ± 0·90 | 0·52 ± 0·05 | 0 | 0·56 | 0·0583 |

air segmentation, while longer wash times should be used in a system without intersample air segmentation. This provides a guideline for an operator who can choose a proper ratio of sample times to wash times, which is based on the presence or absence of intersample air segmentation in his or her analytical system to obtain an optimal result with minimal carry-over at a given sample throughput.

That the precision of analysis generally increases as cycle time increases is indicated by the decrease in the relative standard deviations (RSD) shown in tables 1 and 2. At a given cycle time, the effect of intersample air segmentation on the precision of analysis is shown in figure 2. Removal of the intersample air segmentation not only increases the carry-over coefficients, but also reduces the precision of the analysis at a given sample time. At a given cyle time, RSD decreases as sample time increases. The decrease in RSD is more pronounced in a system without intersample air segmentation than one with intersample air segmentation as shown in figure 2.

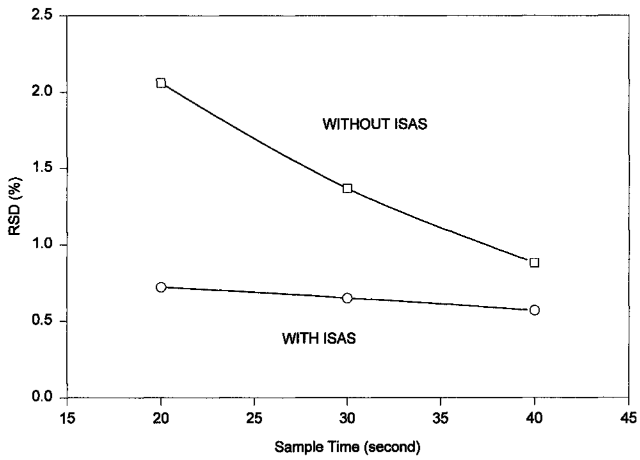

*Figure 2. Comparison of RSD as a function of sample time at a cycle time of 60 s in a system with intersample air segmentation and without intersample air segmentation.*

Carry-over coefficients, $k_{co}$, measured by HLLL and LLHL agree within the standard deviation of the measurements. However, $k_{co}$ values measured by the scheme of LHHH are not in good agreement with the results obtained from the other two schemes. At longer cycle times, some measurements even produced negative values for the carry-over coefficients. It should also be noted that the standard deviations of measurement in the

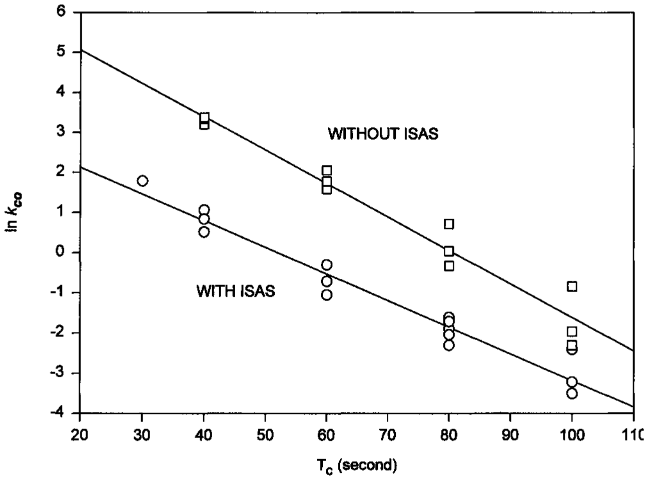

*Figure 3. Comparison of carry-over coefficient as a function of cycle time in a system with intersample air segmentation and without intersample air segmentation.*

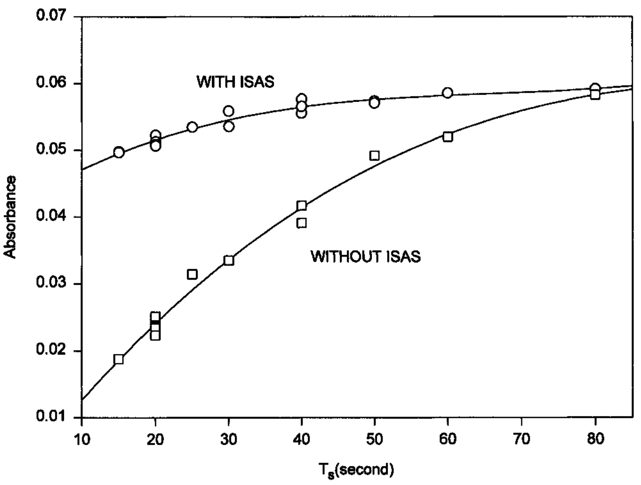

*Figure 4. Comparison of normalized absorbance as a function of sample time in a system with intersample air segmentation and without intersample air segmentation.*

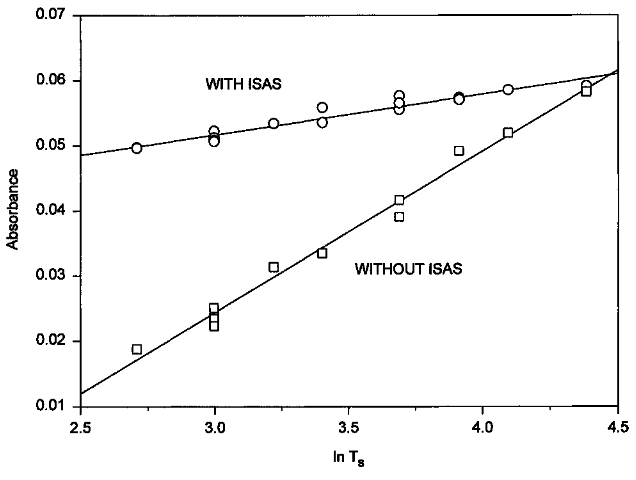

*Figure 5. Linear relationships between normalized absorbance and natural logarithm of sample time in a system with intersample air segmentation and without intersample air segmentation.*

LHHH scheme are significantly greater than that in the other two schemes. The carry-over coefficients, $k_{co}$, are calculated from relative difference of absorbances in consecutive samples with same analyte concentrations. If the random error of analysis, expressed as RSD, is larger than the carry-over coefficient, $k_{co}$, to be measured, then sequential measurements cannot give reliable results. Thus, using HLLL or LLHL schemes, and using blanks as low concentration samples, gives more reliable measurements of $k_{co}$ than using the LHHH scheme.

At a shorter (40 s) cycle time, the carry-over coefficient, $k_{co}$, in a system without intersample air segmentation is 10 times greater than that in a system with intersample air segmentation. The differences become less at longer cycle time (five times greater at a cycle time of 100 s). Carry-over coefficients, $k_{co}$, are a function of cycle time, and the ratio of sample time to wash time as shown in figure 3, and fitted to following equations:

$$\ln k_{co} = 7{\cdot}131 - 0{\cdot}090\,94 T_c + 0{\cdot}4(R - 1) \text{ without ISAS} \quad (34)$$

$$\ln k_{co} = 3{\cdot}724 - 0{\cdot}070\,38 T_c - 0{\cdot}5(R - 1) \text{ with ISAS} \quad (35)$$

where $T_c$ is the cycle time in s and $R$ the ratio of sample time to wash time. These equations can be used to predict carry-over coefficient $k_{co}$ at a given sample time and wash time. They can also be used to select maximum sample throughput with a tolerable carry-over.

The measured absorbances at a given concentration of silicate can be used to quantify the dispersion of a continuous flow system. Tables 1 and 2 list normalized absorbances (to 100 μM silicate) measured at different sample and wash times. The absorbances increased as sample time increased to a constant value of *ca.* 0·06 as shown in figure 4. At shorter sample times the flow system without intersample air segmentation produced much lower absorbances than the system with intersample air segmentation. The differences in absorbances between the two systems become smaller as sample time increases.

At a sample time of 80 s, there was no significant difference between the two systems. The normalized absorbances are a function of the natural logarithm of sample time, as shown in figure 5.

$$\text{absorbance} = 0{\cdot}032\,96 + 0{\cdot}006\,252 \ln T_s \text{ without ISAS} \quad (36)$$

$$\text{absorbance} = 0{\cdot}050\,51 + 0{\cdot}024\,84 \ln T_s \text{ with ISAS} \quad (37)$$

where $T_s$ is the sample time in s. These equations can be used to predict absorbance at a given sample time in a system both with and without intersample air segmentation. The ratio of absorbances in systems with and without intersample air segmentation can be used to estimate the effect of intersample air segmentation on sample dispersion. The ratio of absorbances decreased as sample time increased, figure 6. The ratio is fitted to a second degree function of a natural logarithm of the sample time as follows:

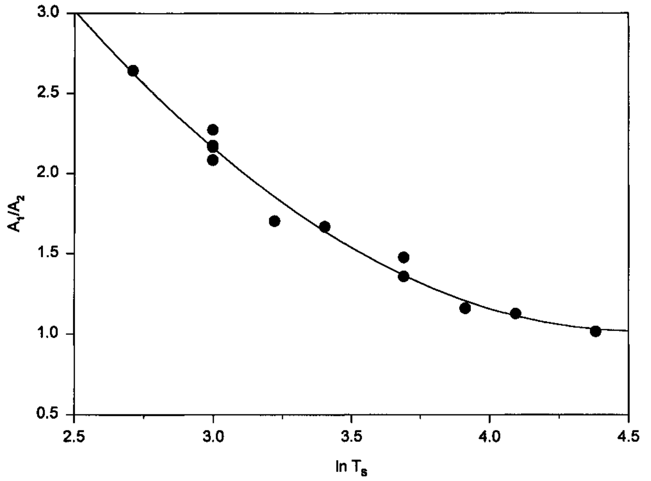

*Figure 6. Ratio of normalized absorbances as a function of natural logarithm of sample time.* $A_1$ *and* $A_2$ *denote absorbances measured from a system with intersample air segmentation and without intersample air segmentation, respectively.*

$$A_1/A_2 = 10{\cdot}93 - 4{\cdot}368 \ln T_s + 0{\cdot}4813 (\ln T_s)^2 \quad (38)$$

where $A_1$ and $A_2$ denote absorbances measured from a system with intersample air segmentation and without intersample air segmentation, respectively. Figure 6 shows that intersample air segmentation greatly reduces sample dispersion. With intersample air segmentation, a sample time of 20 s produced an absorbance that required a sample time of 60 s in a system without intersample air segmentation. Dispersion mainly results from the mixing between sample and wash solution, since sample interaction is negligible under most conditions.

The results presented explain the role of intersample air segmentation in reducing carry-over, sample interaction and dispersion. Unfortunately, intersample bubbles are often removed from a certain portion of the flow stream due to mechanical or chemical limitations. For example, a column packed with copperized cadmium fillings, or granules, is commonly used for the reduction of nitrate to nitrite in nitrate analysis. This device does not permit gas bubbles free passage. Removal of segmentation gas bubbles (including intersample air bubbles) results in enhanced mixing between sample and wash solution. The open tubular cadmium reactors were designed to replace the packed column [17], these permit the segmented gas bubbles to pass through a hollowed cadmium tube and minimize carry-over and dispersion. Intersample air bubbles, however, must be removed to avoid the oxidation of cadmium by oxygen in the air.

Using a debubbler before the flow cell to eliminate bubble-induced interference with the absorbance measurement is also common practice in gas segmented continuous flow analysis. To reduce carry-over, sample interaction and dispersion, a debubbler can be replaced by alternative techniques. Electronic bubble gating has been used in some autoanalysers [18,19]. With a high sensitivity detector, flow cells with volumes smaller than that of a single liquid segment are practicable. This permits the use of a data acquisition program which only retains the signal when the flow cell is completely filled with the liquid segment and filters out the signal while the gas bubbles pass the flow cell. This technique has the potential to greatly reduce carry-over, sample interaction and sample dispersion, because it allows the whole flow stream to remain gas segmented.

### Conclusions

The scheme of LLHL proposed by Thiers *et al.* [5] has been verified theoretically and is recommended for the determination of the carry-over coefficient. It has been suggested that blank solutions should be used as low concentration samples. Carry-over should be determined in at least seven replicates to obtain statistically significant coefficients. The LLLH scheme has been proposed for determination of the sample interaction coefficient. Both HLLL and LHHH measure the sum of the carry-over coefficient and sample interaction coefficient. HLLL can be used to determine carry-over coefficients only under conditions of negligible sample interaction. LHHH has a greater standard deviation and is not recommended.

Experimental results indicate that carry-over is a strong function of cycle time and a weak function of the ratio of sample time to wash time. Normalized absorbances, a measure of sample dispersion, were found to be a function of sample time. For a given system, fitted equations can be used to predict carry-over, absorbance and dispersion and to design optimal operation procedures. Intersample air segmentation has been shown to play an important role in reducing carry-over, sample interaction and sample dispersion. Unless it is absolutely necessary and no alternative approach will suffice, using a debubbler to remove the intersample air segmentation should be avoided.

### Acknowledgements

This work was supported by USEPA (DW13936152-01-0) and NOAA's climate and global change programme. Improvement of the first manuscript by Peter Ortner and the constructive criticism of an anonymous reviewer are gratefully acknowledged.

### References

1. Snyder, L., Levine, J., Stoy, R. and Conetta, A., *Analytical Chemistry*, **48** (1976), 942A.

2. Begg, R. D., *Analytical Chemistry*, **43** (1971), 854.

3. Skeggs, L. T., *American Journal of Clinical Pathology*, **28** (1958), 311.

4. Thiers, R. E. and Oglesby, K. M., *Clinical Chemistry*, **10** (1964), 246.

5. Thiers, R. E., Meyn, J. and Wildermann, R. F., *Clinical Chemistry*, **16** (1970), 832.

6. Thiers, R. E., Cole, R. R. and Kirsch, W. J., *Clinical Chemistry*, **13** (1967), 451.

7. Thiers, R. E., Reed, A. H. and Delander, K., *Clinical Chemistry*, **17** (1971), 42.

8. Broughton, P. M. G., Buttolph, M. A., Gowenlock, A. H., Neill, D. W. and Skentelbery, R. G., *Journal of Clinical Pathology*, **22** (1969), 278.

9. Walker, W. H. C., Pennock, C. A. and McGowan, G. K., *Clinica Chimica Acta*, **27** (1970), 421.

10. Aplkem, SoftPac$^{TM}$ Plus User's Guide, Revision 7/90 (1990).
11. Perstorp Analytical Environmental, FastPac$^{TM}$ Software User's Manual, Version 1.3 (1995).
12. Aplkem, FastPac$^{TM}$ Software User's Manual, Version 1.31 (1996).
13. Bran+Luebbe, TrAAcs and AutoAnalyzer II Operation System Reference Manual, (1996).
14. Angelova, S. and Holy, H.W., *Analytical Chimica Acta*, **145** (1983), 51.
15. Armstrong, F. A. J., Stearns, C. R. and Strickland, J. D. H., *Deep-Sea Research*, **14** (1967), 381.
16. Hansen, H. P., Grasshoff, K., Statham, P. J. and Williams, P. J. LeB. In *Methods of Seawater Analysis*, Eds Grasshoff, K., Ehrhardt, M. and Kremling, K. (Weinheim, Verlag Chemie, Germany 1983).
17. Patton, C. J., Ph.D. dissertation, Michigan State University (1982).
18. Hbig, R. L., Schein, B.W., Walters, L. and Thiers, R. E., *Clinical Chemistry*, **15** (1969), 1045.
19. Patton, C. J., Rabb, M. and Crouch, S. R., *Analytical Chemistry*, **54** (1982), 1113.

